# Crystal Structure of Inhibitor-Bound Bacterial Oligopeptidase B in the Closed State: Similarity and Difference between Protozoan and Bacterial Enzymes

**DOI:** 10.3390/ijms24032286

**Published:** 2023-01-24

**Authors:** Dmitry E. Petrenko, David M. Karlinsky, Veronika D. Gordeeva, Georgij P. Arapidi, Elena V. Britikova, Vladimir V. Britikov, Alena Y. Nikolaeva, Konstantin M. Boyko, Vladimir I. Timofeev, Inna P. Kuranova, Anna G. Mikhailova, Eduard V. Bocharov, Tatiana V. Rakitina

**Affiliations:** 1 National Research Center “Kurchatov Institute”, 123182 Moscow, Russia; 2 Shemyakin-Ovchinnikov Institute of Bioorganic Chemistry of the Russian Academy of Sciences, 117997 Moscow, Russia; 3 Federal Research and Clinical Center of Physical-Chemical Medicine of Federal Medical Biological Agency, 119435 Moscow, Russia; 4 Moscow Institute of Physics and Technology (National Research University), Phystech School of Biological and Medical Physics, 117303 Moscow, Russia; 5 Institute of Bioorganic Chemistry of the National Academy of Sciences of Belarus, 220141 Minsk, Belarus; 6 A.N. Bach Institute of Biochemistry, Federal Research Center of Biotechnology of the Russian Academy of Sciences, 119071 Moscow, Russia; 7 Shubnikov Institute of Crystallography, Federal Scientific Research Centre “Crystallography and Photonics” of the Russian Academy of Sciences, 119333 Moscow, Russia

**Keywords:** serine protease, prolyl oligopeptidase, oligopeptidase B, conformational transitions, catalytic triad, chloromethyl ketone inhibitor, AlphaFold, bioinformatics

## Abstract

The crystal structure of bacterial oligopeptidase B from *Serratia proteamaculans* (SpOpB) in complex with a chloromethyl ketone inhibitor was determined at 2.2 Å resolution. SpOpB was crystallized in a closed (catalytically active) conformation. A single inhibitor molecule bound simultaneously to the catalytic residues S532 and H652 mimicked a tetrahedral intermediate of the catalytic reaction. A comparative analysis of the obtained structure and the structure of OpB from *Trypanosoma brucei* (TbOpB) in a closed conformation showed that in both enzymes, the stabilization of the D-loop (carrying the catalytic D) in a position favorable for the formation of a tetrahedral complex occurs due to interaction with the neighboring loop from the β-propeller. However, the modes of interdomain interactions were significantly different for bacterial and protozoan OpBs. Instead of a salt bridge (as in TbOpB), in SpOpB, a pair of polar residues following the catalytic D617 and a pair of neighboring arginine residues from the β-propeller domain formed complementary oppositely charged surfaces. Bioinformatics analysis and structural modeling show that all bacterial OpBs can be divided into two large groups according to these two modes of D-loop stabilization in closed conformations.

## 1. Introduction

Oligopeptidases B (OpBs, EC 3.4.21.83) are trypsin-like serine peptidases that belong to the prolyl oligopeptidase (POP) family (S9 family) of clan SC [1,2]. Prolyl endopeptidases (named PEPs or POPs, EC 3.4.21.26) and OpBs together form the S9A subfamily. Endopeptidases from the S9A subfamily cleave peptide bonds on the carbonyl side of either proline (POPs) or basic amino acid residues (OpBs). S9B and S9C subfamilies contain dipeptidyl-peptidases IV (DPPs, EC 3.4.14.5) and acyl-aminoacyl peptidases (AAPs, EC 3.4.19.1), respectively. Similar to POPs, exopeptidases DPPs and AAPs are proline-specific. DPPs cleave dipeptides from the N-terminus of oligopeptides, while AAPs remove N-acetylated prolines. The residues of the active center S, D, and H (in accordance with their order in POP sequences) form a classical catalytic triad, where S is a nucleophile, H is a general base and acid, and D participates in the orientation of H residue and neutralization of the charge that is formed on H during transition states.

The main structural characteristic of OpBs, POPs, and the rest of the S9 family is the two-domain architecture [3]. The catalytic domain includes the α/β-hydrolase fold, which is canonical for serine proteases and is located at the C-terminal part of the molecule, and the N-terminal loop, which is divided from the α/β hydrolase fold by the regulatory β-propeller domain (Supplementary Figure S1). The β-propeller domain consists of seven blade-shaped beta sheets arranged around the central axis according to the open Velcro topology (no connection between first and last blades) and restricts access of substrates larger than 3 kDa to the catalytic triad located in the interdomain cavity [4]. These domains are linked by two hinge regions connecting the β-propeller domain with the N-terminal and C-terminal parts of the catalytic domain.

Such a topology is usually associated with characteristic molecular dynamics involving the convergence/divergence (closing/opening) of domains in addition to intradomain rearrangements [5,6,7,8]. These two types of movements cause transitions between open, closed, and intermediate conformations. In the closed (catalytically active) state, the domains and residues of the catalytic triad are located close to each other, allowing the catalysis to proceed. In the open (inactive) state, the domains and residues of the catalytic triad are separated, which prevents the catalysis, but facilitates entering of the substrate into the active site buried in the interdomain cleft. The intermediate state combines the disrupted catalytic triad of the open state with the domains’ closure resembling the closed state.

The open state was detected in the crystals of ligand-free bacterial POPs from *Sphingomonas capsulata* [5] and *Aeromonas punctata* (ApPOP) [6], as well as in crystals of protozoan OpB from *Trypanosoma brucei* (TbOpB) [7]. In addition, the small angle X-ray scattering (SAXS) showed that bacterial OpB from *Serratia proteamaculans* (SpOpB) adopts the open state in solution [8]. The closed state was observed in the crystals of inhibitor-bound POPs from bacteria *Myxococcus xanthus* [5] and ApPOP [6], as well as in those of antipain-bound TbOpB [7] and OpB from *Leishmania major* (LmOpB) [9]. At the same time, bacterial POP from *Microbulbifer arenaceous* [10], as well as mammalian POPs from *Sus scrofa* (SsPOP) [4] and *Homo sapiens* [11], crystallize in the closed state regardless of the substrate/inhibitor binding. Nevertheless, the computational study based on the combination of normal mode analysis and molecular dynamics showed that the domains’ closure and opening identical to those of bacterial POPs are possible for mammalian enzymes [12]. The existence and importance of the interdomain dynamics was confirmed by ^15^N relaxation NMR-experiments [13] and engineering of the artificial disulfide bridges [14], respectively, performed on mammalian SsPOP. The intermediate state was firstly found in the structure of archaeal POP from *Pyrococcus furiosus* (PfPOP) carrying the non-hydrolysable substrate analog prolyl-proline in the interdomain cavity [15]. Then, using both X-ray diffraction and SAXS analysis, we showed that SpOpB adopted the intermediate state in the presence of polyamine spermine [8].

In contrast to POPs, which are presented in all domains of life, OpBs are found only in bacteria and parasitic protozoa [16]. OpBs are known virulence factors of protozoan infections and are viewed as putative targets for treatment of leishmaniasis and trypanosomiasis and/or development of the corresponding vaccines [17,18,19,20]. In addition, bacterial OpBs play an important role in the resistance to non-lytic proline-rich antimicrobial peptides, which are usually enriched with basic amino acid residues [21]. Nevertheless, the restricted distribution of OpBs caused a delay in their structural studies: spatial structures were solved only for two protozoan enzymes (TbOpB and LmOpB) [7,9] and for modified (mutated) derivatives of SpOpB in the intermediate state [8,22,23]. At the same time, numerous structures of both inhibitor-bound and free POPs from nearly a dozen species, including those from mammals, bacteria, archaea, fungi, and mollusks, were solved [4,5,6,10,11,15,24,25].

A mechanism of the catalytic activation common for OpBs, POPs, and AAPs was suggested in [7] based on a comparative structural analysis of the enzymes in the open and closed states. According to [7], for both representatives of the S9A subfamily (OpBs and POPs), the synchronism of domain rearrangement and assembly/disassembly of the catalytic triad was regulated by formation and deformation of a conservative interdomain salt bridge (SB), which was named SB1 in TbOpB. This SB1 links E172 of the β-propeller domain and R650 of the catalytic domain of the TbOpB and stabilizes the neighboring catalytic D648 in the position favorable for catalysis. These residues (E172 and R650 in TbOpB) were conserved in protozoan OpBs and known bacterial POPs [7]. Moreover, their substitutions led to a significant loss of TbOpB catalytic activity [26].

It is intriguing that the residues forming this SB were not conserved in well-studied γ-proteobacterial OpBs from *Escherichia coli*, *Salmonella enterica*, and SpOpB, which carry E to R and R to Q substitutions in the corresponding positions [27].

Here we perform a bioinformatics analysis of more than three thousand sequences of bacterial OpBs and show that the same substitutions were characteristics of more than half of bacterial OpBs (a SpOpB-like group). To solve the puzzle of how the catalytic triad is stabilized in the closed conformation of SpOpB-like enzymes, we obtained and characterized the crystal structure of SpOpB in the closed state. Comparative structural analysis shows that two alternate modes of stabilization of the catalytic triad in the closed conformations of OpBs coexist in the bacterial kingdom.

## 2. Results

### 2.1. Search for the Salt Bridge (SB1) of TbOpB in Protein Sequences of Bacterial OpBs

Amino acid sequences of bacterial OpBs (EC: 3.4.21.83) were acquired from the UniProt Knowledgebase (release 2021_03). After filtering unwanted entries from non-bacterial, metagenome, and environmental samples, a filtered array of 3243 sequences was included in the comparative analysis. The resulting multiple sequence alignment (MSA) of bacterial OpB sequences (Supplementary File S1) was used to search for the protozoan SB1. According to [7], the interdomain SB1 between Q650, located in the same loop with the catalytic residue D648 (D-loop), and R172, located on the opposite loop of the β-propeller, ensures the correct (optimal for the catalytic reaction) orientation of the entire D-loop and, in particular, the catalytic D648, which, in turn, contributes to the correct orientation of the catalytic H. As a result, it was concluded that it is SB1 that stabilizes catalytic triads of both TbOpB and similarly arranged POPs in closed conformations [7].

We postulated that this SB1 requires amino acid residues E (or alternative D) and R in fixed positions on the OpB sequences, namely in the D-loop and in the opposite loop of the β-propeller domain, respectively, calling this amino acid pattern the E/D-R combination. Knowing that in SpOpB these positions are occupied by the residues R151 and Q619, we called this second pattern the R-Q combination.

**Figure 1:**
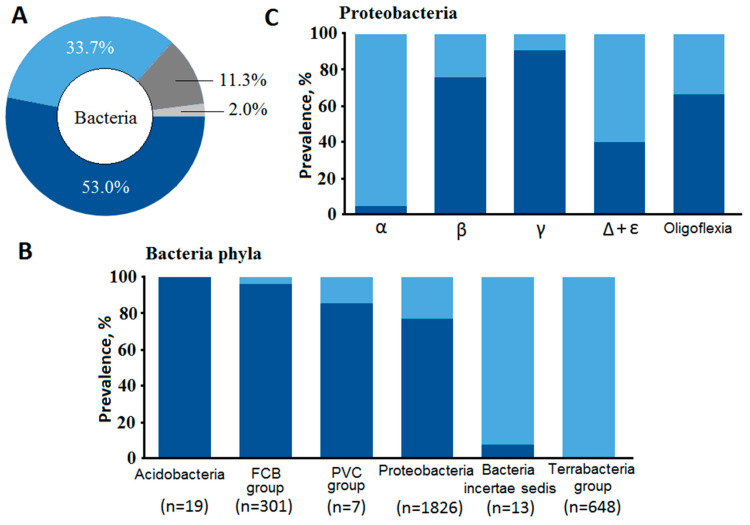
Protozoan SB1 is absent in more than half of bacterial OpB sequences. (**A**) Frequency of occurrence of amino acid combinations R-Q and E/D-R in bacterial OpB sequences are shown for the entire bacterial kingdom (**A**), at the level of superphyla (**B**), and inside the superphylum of Proteobacteria (**C**). Dark blue color corresponds to the combination of amino acids R-Q, light blue colors—E/D-R. Dark and light gray colors are for sequences with other combinations of amino acids in the corresponding positions and short sequences, respectively. In (**B**), Acidobacteria denotes a group of Fibrobacteres/Acidobacteria.

We found the combination of amino acids E/D-R in about 34% of bacterial OpBs (a TbOpB-like group), while the combination of R-Q in the corresponding positions (SpOpB-like group) dominated among bacterial OpBs (Figure 1A). More than 53% of OpB sequences fell into the SpOpB-like group. Only 2% of the sequences contained combinations other than E/D-R and R-Q in the corresponding positions, while short (less than 500 amino acid residues) sequences that did not cover all the studied positions accounted for 11% of the array.

We evaluated the distribution of E/D-R and R-Q combinations in various taxonomic groups (superphylum) of Bacteria, as well as in various classes of the most numerous group of Proteobacteria (Figure 1B,C). In Terrabacteria superphylum and in Bacteria incertae sedis, we observed mainly TbOpB-like sequences. In Acidobacteria and FCB group, we observed mainly SpOpB-like sequences. Proteobacterial OpBs have both variants. The E/D-R combination is predominant in Alphaproteobacteria, while R-Q is predominant in Gammaproteobacteria, the major class of Proteobacteria. The distribution of the corresponding combinations at lower taxonomic levels is shown on the phylogenetic tree (Supplementary Figure S2), which was constructed after exclusion of conflicting data (about 13% of sequences, according to Figure 1A).

**Figure 2:**
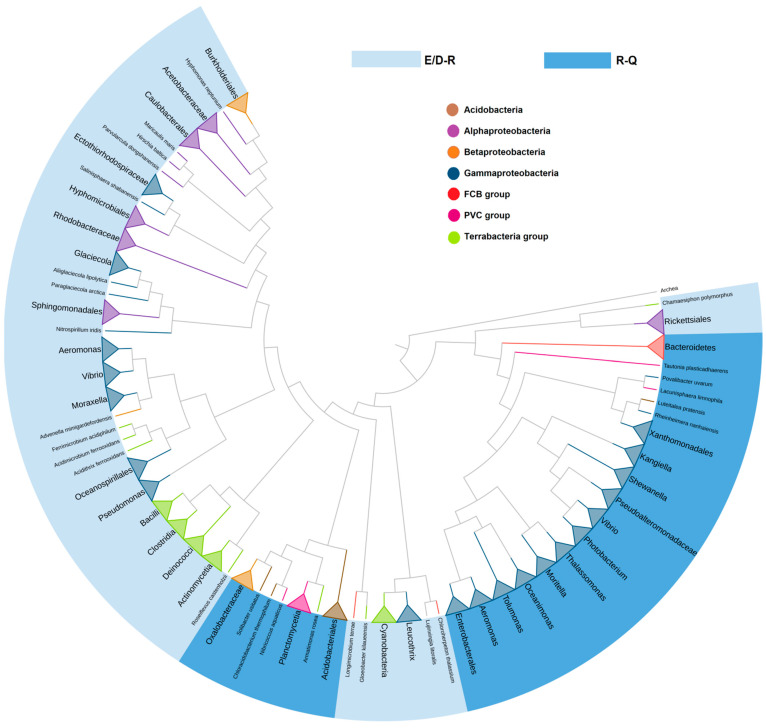
Two independent clusters of SpOpB-like sequences carrying the R-Q amino acid combination are found by a phylogenetic analysis of 1364 bacterial OpB sequences.

To analyze, in more detail, the representation of E/D-R and R-Q combinations at different levels of taxonomy, a phylogenetic analysis of 1364 bacterial OpB sequences from the most representative taxonomic groups was carried out (Figure 2). According to Figure 2, in most cases, the clustering corresponded to the phylum. In addition, two independent clusters can be observed: one for Bacteroidetes and some families of Gammaproteobacteria, the second is for Acidobacteria, PVC group, and Betaproteobacteria.

Good separation and high representativeness of both SpOpB- and TbOpB-like groups prompted us to look for other differences besides the combinations of E/D-R and R-Q used for the initial separation. To search for additional conservative patterns, we prepared 61 taxonomy-based consensus sequences and performed MSA (Supplementary File S2). The sequences of TbOpB (Q382P7), LmOpB (Q4QHU7), and SpOpB (B3VI58) were also included in the analysis.

**Figure 3:**
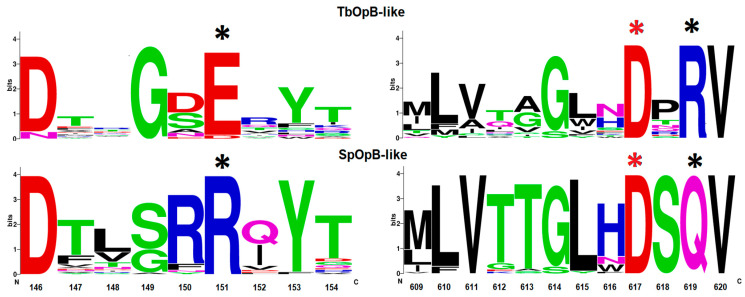
Logo-plot of amino acid patterns surrounding the initial combinations of E/D-R and R-Q in the TbOpB- and SpOpB-like groups, respectively. The height of the letters is proportional to the frequency of occurrence of the corresponding residue. Residues that promote or prevent the formation of a salt bridge are marked with black asterisks, catalytic D, with red asterisks. The numbering corresponds to the SpOpB sequence.

We found that in SpOpB and in the SpOpB-like group, the R-Q combination was associated with highly conserved residues R150 and S618 at positions preceding R151 and Q619, respectively (Figure 3). In addition, less conserved residues T613 and Q621 (numbering according to the SpOpB sequence) were located at the end of the β38-strand and at the beginning of the α11-helix that surrounds the loop containing the catalytic residue D617 (D-loop); while in the TbOpB-like group, residues in the corresponding positions have not been preserved (Figure 3).

The crystal structure of SpOpB in a closed conformation was used to elucidate the structural role of the conservative amino acid pattern found in the SpOpB-like group of bacterial OpBs.

### 2.2. Comparative Structural Analysis of Bacterial SpOpB and Protozoan TbOpB in Various Conformations

#### 2.2.1. Structural Overview of TCK-Bound SpOpB

The structure of wild type SpOpB bound to tosyl-L-lysine chloromethyl ketone (TCK) was determined at 2.2 Å resolution. The polypeptide chain contains 685 amino acid residues, nine of which, including the N-terminal His-tag (MASHHHHHH), were not detected in the electron density. The unit cell dimensions of the SpOpB-TCK crystal were 75.529 Å × 89.660 Å × 108.650 Å, a crystallographic space group was P2(1)2(1)2(1), an asymmetric unit contained a monomer in a crystal lattice.

Previously, crystal structures were obtained for the following mutated forms of SpOpB: catalytically inactive SpOpB_S532A, carrying the alanine substitution of the catalytic residue S532 (PDB ID 7ZJZ), and low-active enzymes SpOpBmod, in which the first hinge peptide (residues 71-77, IPQQEH) was replaced by the tobacco etching virus protease site (ENLYFQ) [8,22,23]. SpOpBmod was crystallized in both the inhibitor-free form (PDB ID 7OB1, 7YWS, and 7YX7) and in the TCK-bound form (PDB ID 7NE7). All mutated forms of SpOpB were crystallized in intermediate conformations and the corresponding crystals have unit cell dimensions slightly increased compared to the SpOpB-TCK crystal: 70.71 Å × 100.4 Å × 108.67 Å (SpOpB_S532A) and 73.32 Å × 101.1 Å × 108.76 Å (SpOpBmod-TCK). The reduced volume of the unit cell indicates an increased proximity of the domains, which, in turn, suggests that SpOpB-TCK crystallizes in different conformation compared to the previously reported structures.

A clear electron density for one molecule of covalently bound TCK, which is a modified lysine residue tosylated at the N-terminus and chloromethylated at the C-terminus, was observed from the *2mFo-DFc* difference map in the active site (Supplementary Figure S3A). A single inhibitor molecule was bound simultaneously to the S532 and H652 residues of the catalytic triad. Despite the presence of spermine in the crystallization mixture, no spermine molecules were found in the crystal structure.

#### 2.2.2. TCK-Bound SpOpB Crystallized in the Closed Conformation

**Figure 4:**
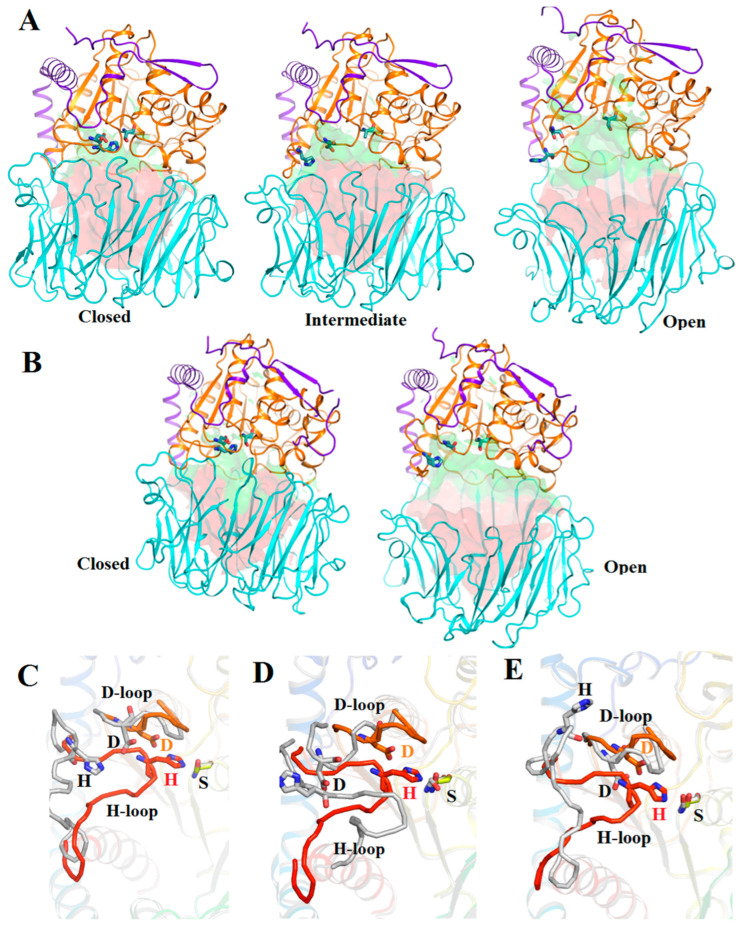
Visualization of the main structural characteristics of SpOpB and TbOpB in various conformations. (**A**) Cartoon/surface presentations of the structures of SpOpB-TCK in the closed state (PDB ID 7YWP), SpOpB_S532A in the intermediate state (PDB ID 7ZJZ), and SAXS-validated model of SpOpB in the open state. The N-terminal loops of the catalytic domains are in violet, the β-propeller domains are in cyan, and the α/β-hydrolase folds are in orange. The catalytic triad residues are shown in emerald sticks; the inhibitor molecule in the 7YWP structure is omitted. The solution accessible areas in the funnel-like β-propeller tunnel and interdomain cavity are colored in red and green, respectively. (**B**) Similar presentations of the TbOpB structures in the closed and open states (PDB ID 4BP9 and 4BP8, respectively). (**C**,**D**) The assembled catalytic triad in the SpOpB closed state (rainbow coloring) superimposed on the disrupted catalytic triad (colored in grey) in the intermediate (**C**) and open (**D**) conformations of SpOpB. The catalytic residues are shown as sticks and named; the covalently bound inhibitor is omitted. (**E**) Similar superposition of the assembled and dissembled catalytic triad in the closed and open state of TbOpB.

We compared the obtained structure of SpOpB-TCK with the SpOpB_S532A structure in the intermediate conformation and SAXS-validated model of SpOpB in the open state (SpOpB-SAXS) reported in [23], as well as with structures of protozoan TbOpB in the open (ligand-free) and closed (inhibitor-bound) states (PDB ID 4BP8 and 4BP9, respectively) [7]. Global (interdomain) and local (intradomain) rearrangements associated with different conformations of SpOpB and TbOpB are shown in Figure 4.

The reduced volumes of interdomain cavities are associated with the convergence (closure) of domains in the closed state compared to the open state of SpOpB and TbOpB (Figure 4A,B). The mutual arrangement of domains in the SpOpB intermediate state is not so different from that in the closed state compared to the open state (Figure 4A).

In OpBs and POPs, the catalytic triad consists of the amino acid residues S, D, and H (S532, D617, and H652 in SpOpB or S563, D648, and H683 in TbOpB), which create the charge-relay system for the nucleophilic attack by the catalytic S. The catalytic triad assembling/dissembling is mostly a result of the movement of the flexible loop carrying a catalytic H residue (H-loop) (Figure 4C–E). Due to the rearrangement of the H-loop, the catalytic H shifts from the surface to the center of the interdomain cavity, where it is embedded between two other residues of the catalytic triad, while the positions of the catalytic D and S change less significantly and do not change at all, respectively.

Quantitative analysis of the mutual arrangement of the catalytic triad residues and domains in the different conformations of SpOpB and TbOpB was performed using the approach based on the combination of the Dali [28] and PDBePisa [29] internet services (Table 1).

According to Table 1, at the transition between the closed and open conformations, the shifts of the Cα and NE2 atoms of the catalytic H towards the Cα and OG atoms of the catalytic S are the same for TbOpB and SpOpB and reach 10 and 15 Å or more, respectively. Thus, these distances are obvious differentiating criteria for the assembled and disassembled catalytic triad, which is the main characteristic of the closed and open (or intermediate) state, respectively. The degree of proximity/remoteness of domains can also be quantified using several easily measurable parameters. Based on Table 1, we suggest that both the distances between centers of mass of the domains and the percentage of buried surface area from the total area or the percentage of residues in the interdomain interface are good indicators of the conformational state. According to these criteria, the newly obtained structure of SpOpB-TCK indeed represents the closed state of the enzyme.

A detailed analysis of the changes in interfaces between the catalytic and propeller domains in three conformational states of SpOpB is presented in Table 2. As follows from this table, only a part of the propeller domain, namely blades 1–4 (numbering in the direction from the 1st hinge peptide to 2nd, see Supplementary Figure S1), significantly changes its position relative to the catalytic domain. The positions of blades 5–7 practically do not change, as evidenced by the conservation of the interdomain SBs and hydrogen (H) bonds. Moreover, in the open conformation, additional H-bonds appear in this region, which probably compensates for the complete absence of interdomain contacts in the more mobile half of the propeller domain (blades 1–4), the movement of which opens the entrance to the interdomain cavity and increases the volume of the latter (see Figure 4A). At the same time, a comparison of the closed and intermediate conformations shows that, despite the smaller difference in the volumes of the interdomain cavity compared to the open conformation of SpOpB (Figure 4A), the interfaces between the domains differ significantly (Table 2). The propeller blades 2 and 3 have polar contacts with the catalytic domain only in the closed conformation. The propeller blades 1 and 4 form contacts with the catalytic domain in both closed and intermediate conformations. However, in the closed conformation, numerous contacts, including both SBs and H-bonds, are observed, while in the intermediate, the SBs and the major part of H-bonds are absent, and the rest of the H-bonds are shifted along the contacting surfaces (Table 2).

#### 2.2.3. The TCK-Bound SpOpB Catalytic Center Simulates the Tetrahedral Transition State Analogue Complex

The closed state of SpOpB was associated with covalent binding of a single TCK molecule to the catalytic S532 and H652 residues. The binding to the OG atom of S532 occurs through the hemiacetal carbon atom of TCK, and to the NE2 atom of H652, it occurs through the methylene group of TCK (Supplementary Figure S3A). The kinetic parameters of the alkylation reaction between SpOpB and TCK were determined as described in Materials and Methods section. The dissociation constant (*K*_i_) of the enzyme-inhibitor complex and *k*_2_—the second-order rate constant for the alkylation step were 0.28 ± 0.06 mM and 0.27 ± 0.03 min^−1^, respectively. The inhibition of the SpOpB catalytic activity during the titration by TCK is shown in Supplementary Figure S3B.

The observed SpOpB-TCK complex represents the classical mode of interaction of the specific chloromethyl ketone inhibitor with trypsin-like serine protease. The interaction was reported for the peptide inhibitors of trypsin (D-Val-Phe-Lys-CH_2_Cl [30]) and enterokinase (Val-(Asp)_4_-Lys-CH_2_Cl [31]) and was observed in the crystal structure of TCK-bound lysine-specific endoproteinase from *Lysobacter enzymogenes* (PDB ID 4NSY, [32]).

**Figure 5:**
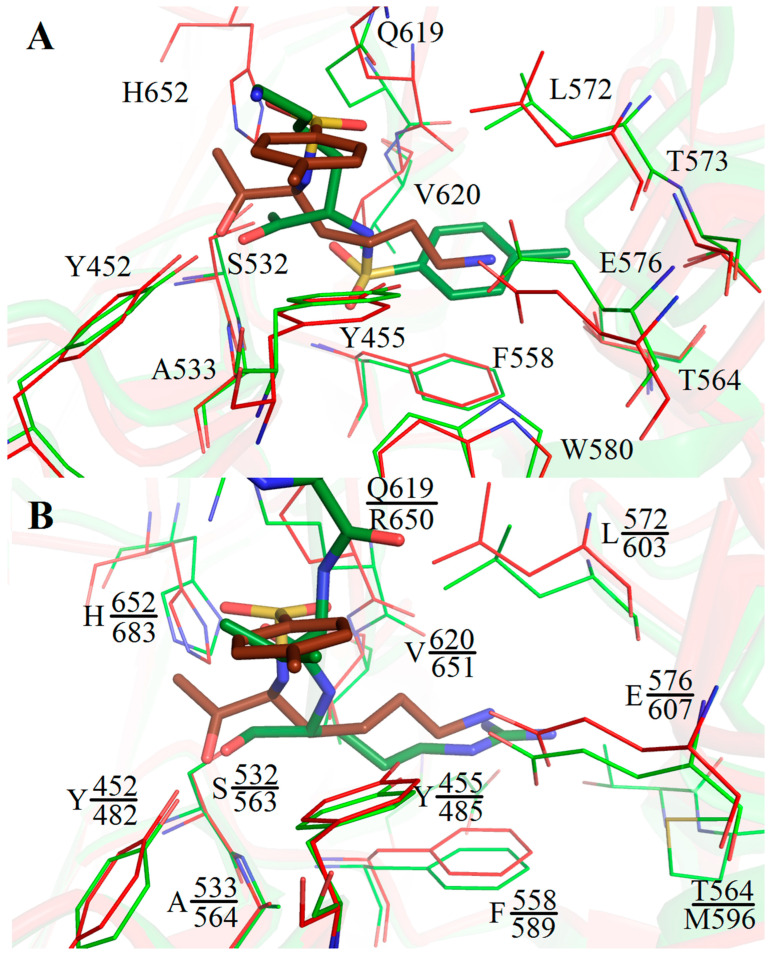
Amino acid surroundings of the covalently bound inhibitors in the SpOpB-TCK structure (PDB ID 7YWP), PSPmod-TCK structure (PDB ID 7NE7), and TbOpB-AIP structure (PDB ID 4BP9). (**A**) Classical position of S532- and H652-bound TCK (colored in brown) in the substrate-binding pocket of SpOpB in the closed state (colored in red) and inversed position of the S532-bound TCK (colored in green) in the substrate-binding pocket of SpOBmod in the intermediate state (colored in light green). (**B**) The Cα-atom superposition of the SpOpB-TCK complex (colored in red and brown) on the TbOpB-AIP complex (colored in light green and green). Amino acid residues in the nearest surroundings (4 Å) of the inhibitors are shown in sticks and signed. Upper labels are for the SpOpB residues, lower ones are for the TbOpB residues. Only P1 arginyl and P2 valine of AIP are shown.

An unusual type of binding was found in the SpOpBmod-TCK structure, in which two catalytic triad residues, S532 and H652, were bound to two different TCK molecules via the TCK methylene groups [23]. Such a type of binding stabilized the enzyme in the intermediate conformation with the dissembled catalytic triad, since the TCK-bound H652 was fixed in the surface of the enzyme [23]. Comparison of the S532-bound TCK molecules in SpOpBmod-TCK and SpOpB-TCK structures shows that the tosyl aromatic ring and the lysine side chain of TCK practically exchange their positions, but this rearrangement does not affect the amino acid surroundings of the inhibitor, which include only residues from the catalytic domain (Figure 5A).

Comparison of TCK position in the SpOpB-TCK structure with that of another covalent inhibitor of trypsin-like serine proteases, transition state analogues, antipain (AIP) in the TbOpB-AIP structure (PDB ID 4BP9) shows that residues forming the S1 and S2 substrate-binding pockets are mostly conserved and occupy similar positions in both structures (Figure 5B).

The position of the lysine side chain of TCK coincides with the position of the P1 arginyl of AIP and the position of the tosyl ring coincides with that of the P2 valine of AIP. Both the lysine amino group of TCK and the arginyl guanidino group of AIP interact with E576 and E607 carboxyl groups of SpOpB and TbOpB, respectively. These glutamates are responsible for the primary substrate specificity, an interaction with the P1 residue of the substrate. In addition, both P1 residues are stabilized via Y455/Y485, V620/V651, and F558/F589 (a side chain–side chain π-stacking interaction) of SpOpB/TbOpB. The oxyanions of the tetrahedral complexes are stabilized via H-bonds with Y452/482OH and A533/564N of SpOpB/TbOpB. The carbonyl group of P2 valine of AIP is H-bound to the R650-NH1 atom of TbOpB, while the similarly located O1S atom of TCK is H-bound to the Q619-NE2 of SpOpB.

The SpOpB-TCK complex can be considered as an analogue of the transition state in the proteolytic reaction catalyzed by S532. This allowed adopting the classical scheme of catalysis by trypsin-like proteases to the SpOpB case. The suggested scheme of the reaction is shown in Supplementary Figure S4.

#### 2.2.4. Catalytic Triad Stabilization in the Closed State of Bacterial SpOpB and Protozoan TbOpB

**Figure 6:**
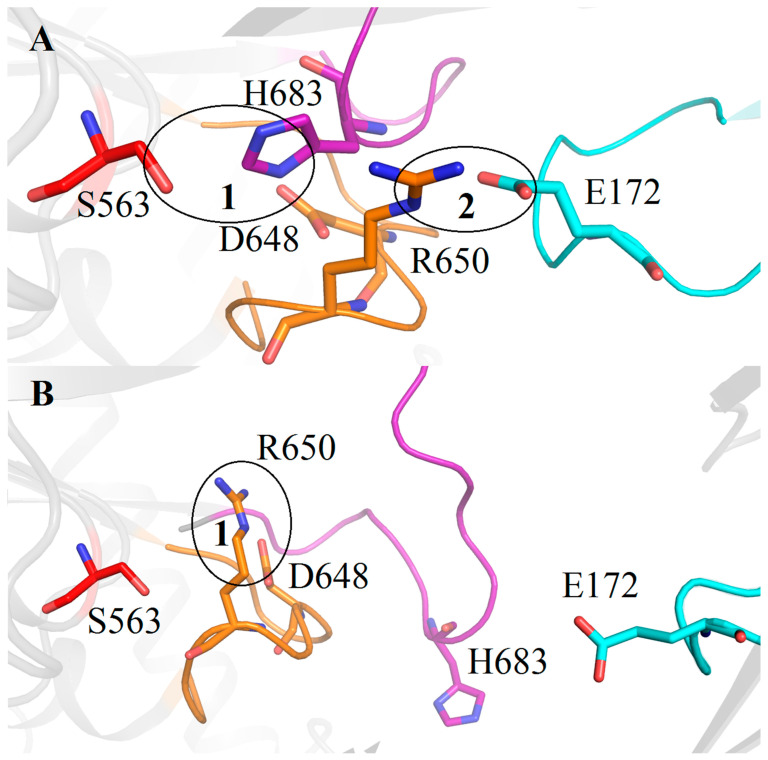
Cartoon/ribbon presentation of the assembled and dissembled catalytic triad in the closed (**A**) and open (**B**) state of TbOpB, respectively. Catalytic residues S563, H683, and D648 are colored in red, magenta, and gold, respectively. The covalently bound inhibitor is omitted. H- and D-loops are in the same color as the respective catalytic residues. The β-propeller loop carrying the E172 residue, which forms the interdomain SB1 with R650 and stabilizes the catalytic triad in the closed conformation, is colored in cyan. The interactions described in the text are encircled and enumerated.

**Figure 7:**
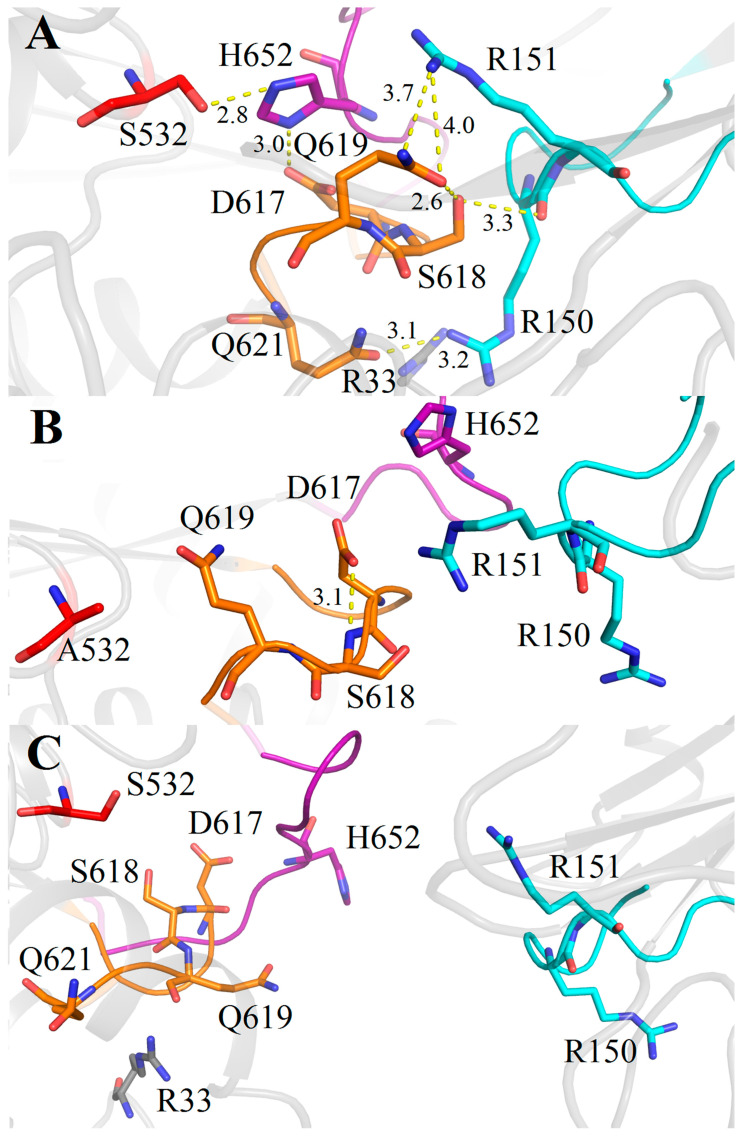
Cartoon/ribbon presentation of the assembled and dissembled catalytic triad in the closed (**A**), intermediate (**B**), and open (**C**) states of SpOpB. Catalytic residues S532, H552, and D617 (together with neighboring residues) are colored in red, magenta, and gold, respectively. The covalently bound inhibitor is omitted. The H- and D-loops are in the same color as the respective catalytic residues. The R150 and R151 pair and the respective β-propeller loop are colored in cyan. All interactions described in the text are shown by yellow dashed lines; distances are given in angstroms.

In both protozoan and bacterial enzymes, represented by TbOpB and SpOpB, respectively, covalent binding of the inhibitor (AIP and TCK) was accompanied by assembling of the catalytic triad, during which, due to the rearrangements of both D- and H-loops, the catalytic H occupies the position between two other catalytic residues (Figure 6A and Figure 7A). As was suggested in [7], local movements in the active site were associated with global interdomain dynamics.

In the TbOpB closed state, catalytic residues S563, H683, and D648 are in the positions favorable for proton transfer from the S563 hydroxyl group to the NE2 atom of H683 and from the ND1 atom of H683 to the D648 carboxyl group (see encircled area 1 in Figure 6A and Table 1 for the corresponding distances). These local arrangements are stabilized by the interdomain SB1 between the E172 residue from the β-propeller domain and the R650 residue from the catalytic domain located near to the catalytic D648 (encircled area 2 in Figure 6A). The distance from the E172-OE1 atom to the R650-NH2 atom is 3.4 Å.

The domains’ opening leads to disruption of the SB1 and freeing the R650 guanidino group for interaction with the catalytic D648 carboxyl group, the D648-OD2—R650-NH2 distance is 2.9 Å (encircled area 1 in Figure 6B). Due to this rearrangement, D648 replaces H683 in the position near catalytic S563, and H683 shifts to the periphery of the catalytic domain [7].

As we discussed above, the residues forming the SB1 in TbOpB are conserved in protozoan OpBs and in approximately 34% of bacterial OpBs, while SpOpB and more than 53% of bacterial OpBs carry the very conservative substitutions (E to R and R to Q). Moreover, in the SpOpB-like group, these substitutions, E172/R151 and R650/Q619 (TbOpB/SpOpB numbering), are associated with the presence of additional conserved residues in the neighboring positions. In SpOpB, R150 and S618 precede R151 and Q619, respectively, while in the TbOpB-like group, residues in the respective positions are not conserved (Figure 3).

We evaluated structural roles of these two pairs of residues (R150-R151 and S618-Q619) in stabilization of the SpOpB catalytic triad. The structures of SpOpB-TCK in the closed state, SpOpB-S532A in the intermediate state, and the SAXS-validated model of SpOpB in the open state were used for analysis. As follows from Figure 7, similar to TbOpB, the rearrangement of the D-loop is observed in the closed state of SpOpB, compared to the intermediate and open states. For example, a comparison of the intermediate and closed states (Figure 7A,B) shows that in the latter state, Q619 rotates around its CG atom, so its CO and NH2 groups replace each other’s positions, while the side chain of D617 rotates almost 180 degrees around its Cα atom. As a result, the Q619 side chain empties the space, which it occupied in the intermediate state, for the H652 side chain. In this position, the NE2 atom of the H652 imidazole ring approaches the Ser532 hydroxyl group and the ND1 atom orientates towards the D617 carboxyl group (see Table 1 for the corresponding distances).

**Figure 8:**
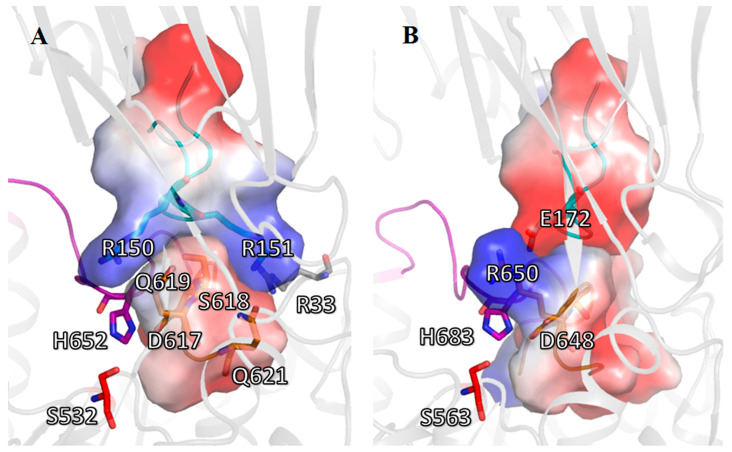
Cartoon/surface presentation of the electrostatic interactions between D-loops of the catalytic domains and the R151/E172-carrying loop (R/E-loop) of the β-propeller domain in the closed state of SpOpB (**A**) and TbOpB (**B**). The H-, D-, and R/E-loops are in magenta, gold, and cyan, respectively. Amino acid residues L615—Q621 (SpOpB) and A644—Y653 (TbOpB) from the D-loops, as well as D146—D154 (SpOpB) and D167—S175 (TbOpB) from the R/E-loops, are shown as surfaces colored according to their electrostatic potential. Positive and negative charges are in blue and red, respectively. The color scale is in units of kT/e ranging from −50 to +50.

Analysis of the polar contacts stabilizing the assembled configuration of the catalytic triad in the closed state of SpOpB shows that the residues from the β-propeller loop, which carries the pair R150-R151, form H-bonds S149OG—G651N (3.1 Å) and R150NH1—Q621OE1 (3.1 Å) with their partners from the H-loop and from the beginning α11-helix bordering the D-loop, respectively. In addition, the R151 side chain and the R150 main chain are orientated toward the side chains of the conserved S618 and Q619 residues. The distances R151-NH1—Q619-NE2, S618-OG—Q619-OE1, and S618-OG—R150-O are 3.7 Å, 2.6 Å, and 3.3 Å, respectively (Figure 7A). Together, R150 and R151 residues form a fork-shaped basic surface that wraps around the adjacent part of the negatively charged D-loop and the adjoining part of the α11-helix (residues D617-S619 and Q621) (Figure 8A). We must mention that the bioinformatics analysis suggested Q621 to be conserved among SpOpB-like group representatives (Figure 3).

In the intermediate and open conformations of SpOpB, all the interactions between the D-loop and the β-propeller domain are broken (Figure 7B,C). During the rearrangement of the catalytic triad in the intermediate state of SpOpB, the H-loop moves to the periphery of the catalytic domain, losing its contact with the D-loop (Table 3). The Q619 side chain replaces the H652 imidazole ring; the D617 carboxyl group forms a H-bond with the N atom of the S618 main chain (Figure 7B), while the O atom of the D617 main chain, together with that of neighboring L615, interacts with the guanidino group of R33 from the N-terminal loop (Table 3).

In the SpOpB open state, the D-loop moves to the periphery of the catalytic domain together with the H-loop, keeping numerous contacts with the latter (Figure 7C and Table 3). The Cα atoms of Q619 and D617 shift to 2.4 and 3.1 Å, respectively. As a result, the carboxyl group of D617 interacts simultaneously with the side chain of R658 from the α12-helix as well as with the main chains of H652 and G653 (Table 3).

Thus, in the assembly/disassembly of the catalytic triad in TbOpB, two residues, E172 and R650, which form a salt bridge when the domains approach each other, play the main role. In SpOpB, this function is divided between the pair of neighboring residues (R150 and R151) from the β-propeller domain and residues from the D-loop and adjoining part of the α11-helix (S618, Q619, and Q621, respectively). These five residues form two oppositely charged complementary areas on the surfaces of the β-propeller and catalytic domains, instead of one interdomain, SB1, observed in TbOpB (Figure 8).

According to structural analysis, there are two modes of interaction between the D-loop of the catalytic domain and the opposite loop from the β-propeller. Both types of interactions fix the position of the D-loops in the closed conformations of OpBs and, consequently, contribute to the orientation of the catalytic D residues favorable for the formation of a tetrahedral complex. Thus, we can say that there are two modes of stabilization of the catalytic triad in TbOpB and SpOpB. In the case of SpOpB, complementary oppositely charged surfaces with a low charge difference are used to stabilize the catalytic triad, while in the case of TbOpB, a point electrostatic contact with a high charge difference (SB1) is used. It can be noted that, in contrast to TbOpB, where both residues forming the SB1 are functionally important [26], in SpOpB, the substitution of R151 did not cause such a strong effect on the catalytic activity [33] because the remaining residues apparently compensated its absence.

### 2.3. Stabilization of the Catalytic Triad in the AlphaFold-Built Models of the SpOpB- and TbOpB-like Oligopeptidases B

We decided to check how the catalytic triads are stabilized in random representatives of bacterial OpBs belonging to either the SpOpB- or TbOpB-like group. For this purpose, we have selected enzymes from bacterial pathogens, which are causative agents of hospital or healthcare-acquired infections, also called nosocomial infections, which spread by various means among susceptible patients in the clinical environment. Nosocomial pathogens cause severe pneumonia or invade the bloodstream and urinary and gastrointestinal tracts. Moreover, due to increased multidrug resistance, they often avoid commonly used antibiotics, but are expected to be susceptible to antimicrobial peptides [34,35]. Hospital-acquired infections are usually associated with six high virulent and antibiotic resistant bacteria: *Enterococcus faecium*, *Staphylococcus aureus*, *Klebsiella pneumoniae*, *Acinetobacter baumannii*, *Pseudomonas aeruginosa*, and *Enterobacter* spp. (ESKAPE group [36]), as well as with *Stenotrophomonas maltophilia*, *Clostridium difficile*, *Mycobacterium tuberculosis*, and several others.

Sequences and structures of OpBs from *K. pneumoniae*, *P. aeruginosa*, *S. maltophilia*, and *M. tuberculosis* were downloaded from the UniProt database and compared with those of SpOpB and TbOpB (Table 4 and Supplementary File S4). No OpBs were found in Gram-positive bacteria *E. faecium*, *S. aureus*, and *C. difficile*, as well as in *A. baumannii* and in several pathogenic representatives of *Enterobacter.* All 3D models used for comparative analysis were built using the AlphaFold program [37] and represented enzymes are in closed conformations, which made it possible to evaluate the mode of stabilization of the catalytic triad.

**Figure 9:**
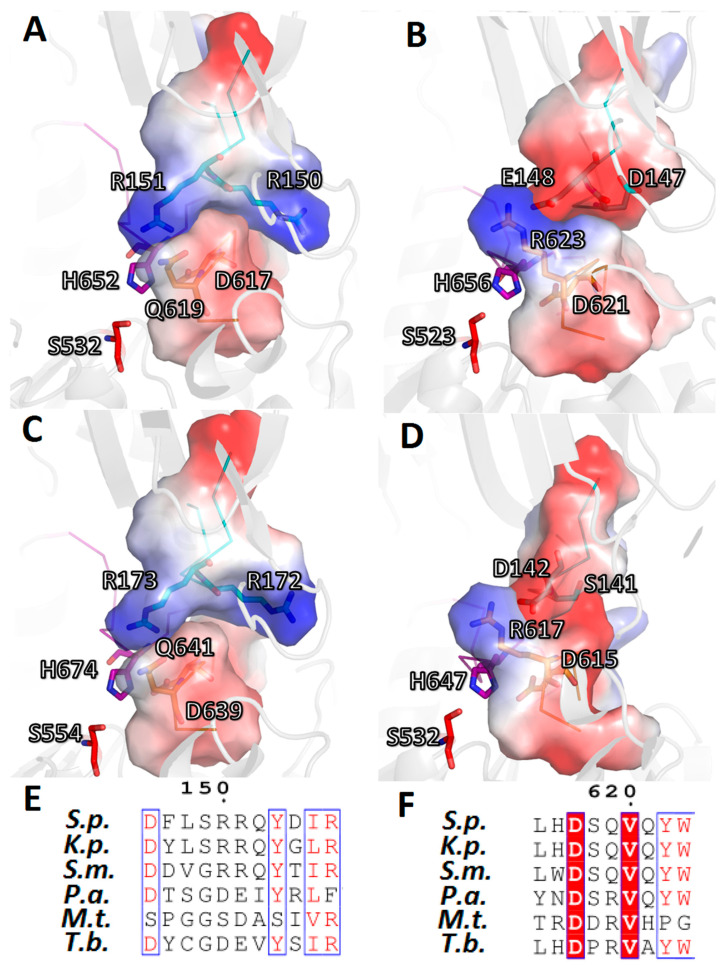
Two modes of stabilization of the catalytic triads in the AlphaFold-built models of bacterial OpBs from nosocomial pathogens. Electrostatic interactions between D-loops of the catalytic domains and its opposite partner-loops (R/E-loops) of the β-propeller domains are shown as cartoon/surface presentations for: (**A**) *K. pneumoniae* (K.p.), (**B**) *P. aeruginosa* (P.a.), (**C**) *S. maltophilia* (S.m.), (**D**) *M. tuberculosis* (M.t.). The H-, D-, and R/E-loops are in magenta, gold, and cyan, respectively. Amino acid residues 615–621 (K.p.), 619–625 (P.a.), 637–643 (S.m.), and 613–619 (M.t.) from D-loops, as well as 146–154 (K.p.), 143–151 (P.a.), 168–176 (S.m.), and 137–145 (M.t.) from R/E-loops, are shown as surfaces and colored according to their electrostatic potential. Positive and negative charges are in blue and red, respectively. The color scale is in units of kT/e ranging from −50 to +50. (**E**,**F**) The amino acid alignments of R/E- and D-loops, respectively. Highly conserved residues are highlighted in red, semi-conserved are colored in red. The corresponding sequences from SpOpB (S.p.) and TbOpB (T.b.) are included for comparison. Numbering is according to the SpOpB sequence.

We found out that OpBs from *K. pneumonia* and *S. maltophilia* carry the SpOpB-like R-Q combination, which, after our bioinformatics and structural studies, was refined as an RR-SQ pattern. Similar to SpOpB (Figure 8A), this RR-SQ pattern causes formation of complementary, oppositely charged surfaces between D-loops and their opposite partner-loops of the β-propeller domains (Figure 9A,C). In turn, OpBs from *P. aeruginosa* and *M. tuberculosis* carry the TbOpB-like E/D-R amino acid patterns, which promote formation of the interdomain SBs (Figure 9B,D).

## 3. Materials and Methods

### 3.1. Bioinformatics Study

Amino acid sequences and taxonomic annotations of OpBs (EC: 3.4.21.83) were downloaded from the UniProt database [38]. Sequences with uncertain taxonomy classification of the source organism were excluded. Multiple sequence alignment was performed using Clustal Omega v.1.2.1 [39]. Results were visualized using Jalview software [40]. A maximum likelihood phylogenetic tree was constructed using the Fast Tree program with the Whelan Goldman model of amino acid evolution [41].

Consensus sequences for each taxonomic group were created using EMBOSS Cons. [42]. A hierarchical tree was constructed using information about taxonomic linkage for each protein sequence. Sequences of the same type (SpOpB-like or Tb-OpB-like) were linked to a higher level.

### 3.2. Production of a Recombinant Protein

SpOpB-expressing plasmid (pET-6HisOpB) was obtained as described in [43]. Expression of the recombinant protein was carried out in *E. coli* BL21(DE3)RIPL (Novagen, Madison, WI, USA). Freshly transformed cells were grown in high salt LB medium with 100 µg/mL ampicillin and 34 µg/mL chloramphenicol (Panreac-AppliChem, Darmstadt, Germany) at 37 °C, until the OD_600_ value reached 0.8. Then, expression was induced with 0.2 mM IPTG. After 20 h incubation at 25 °C, the cells were harvested by centrifugation, resuspended in (50 mM TrisHCl and 500 mM NaCl, pH 8.0 buffer supplemented with 0.1% (*v*/*v*) Triton X-100, 10% (*v*/*v*) glycerol, 20 mM imidazole, and 1 mM PMSF), and disrupted by sonication. After centrifugation (20,000× *g*, 30 min, 4 °C), the supernatant was filtered and applied to a 5 mL HisTrap HP column (GE healthcare, Chicago, IL, USA) equilibrated with the same buffer. The column was washed with Tris/NaCl buffer pH8.0 supplemented with 50 mM imidazole, and SpOpB was eluted by Tris/NaCl buffer pH8.0 supplemented with 300 mM imidazole. The 30 kDa cutoff centrifugal filter devices (Millipore, MA, USA) were used for buffer exchange and protein concentration. Protein size, purity, and oligomeric state were controlled by electrophoresis in SDS-PAAG (Supplementary Figure S5). Protein concentration was determined by the Bradford method.

### 3.3. Determination of the SpOpB Catalytic Activity and Kinetic Parameters of Its Inhibition by TCK

The catalytic activity of SpOpB in presence and in absence of an inhibitor was monitored by hydrolysis of Nα-benzoyl-D,L-arginine-p-nitroanilide (BAPNA) (Sigma-Aldrich, St. Louis, MI, USA), as described in [22]. In brief, we measured the increase in the absorption at 405 nm (0.1 M Tris-HCl, pH 8.0, 2% DMSO, 25 °C), which occurred due to the formation of free p-nitroaniline (Δε405 = 10,400 M^–1^·cm^–1^). Stock solution of BAPNA (20 mM) was prepared in DMSO.

An alkylation reaction between SpOpB (E) and TCK (I) was performed in 200 µL of 0.1 M Tris-HCl buffer, (pH 8.0) containing PSP (70 nM) and TCK (50–260 µM), at 25 °C. At selected time intervals, 30 µL-samples were removed and residual activity of the enzyme was measured. At least three independent experiments for each concentration of TCK were performed.

The kinetic parameters of the alkylation reaction between E and I were determined as described by Kitz and Wilson [44] for irreversible enzyme inhibition under pseudo-first order conditions ([I] ≫ [E]), including the alkylation of enzymes with trypsin-like specificity by chloromethyl ketone inhibitors [30,31].

For each TCK concentration, the pseudo first-order rate constant for inactivation, *k*′, was determined from the plots of relationship ln[E] = *−k*′t + ln[E_0_], where [E] is the concentration of the active enzyme remaining at time (t) and [E_0_] is the initial or total concentration of the enzyme. The inactivation reaction was described by the equation:

$$E+I\;\overset{K_{i}}{↔}\;\mathrm{EI}\;\overset{k_{2}}{\to }\;\mathrm{EI}\prime ,$$

where *K*_i_ is the dissociation constant of the enzyme-inhibitor complex and *k*_2_ is the second-order rate constant for the alkylation step [30,31,44].

In this case, the pseudo first-order rate constant (*k*′) for irreversible inhibition depends on the concentration of the inhibitor according to the Michaelis–Menten-type equation. From a plot of the inverse of the apparent pseudo-first order rate constant (*k*′) vs. the inverse of the concentration of the inhibitor ([I]): 1/*k′* = (*K*_i_/*k*_2_)(1/[I]) + 1/*k*_2_, (Supplementary Figure S3) the kinetic constants of inhibition were obtained.

### 3.4. Crystallization of the SpOpB-TCK Complex

SpOpB was concentrated to 20.0 mg/mL in the buffer (20 mM TrisHCl, pH 8.0, 100 mM NaCl). We prepared 100 mM stock solution of TCK in water. TCK was added to SpOpB dropwise with constant stirring at +4 °C until the final concentration of 1 mM (stoichiometric ratio protein:inhibitor was about 1:4). The loss of the catalytic activity was monitored by hydrolysis of Nα-benzoyl-D,L-arginine-p-nitroanilide (BAPNA) (Sigma-Aldrich, St. Louis, MI, USA) as described in [22]. In brief, we measure the increase in the absorption at 405 nm (0.1 M Tris-HCl, pH 8.0, 2% DMSO, 25 °C), which occurred due to the formation of free p-nitroaniline (Δε405 = 10,400 M^–1^·cm^–1^). Stock solutions of BAPNA (20 mM) were prepared in DMSO.

The excess of inhibitor was removed by size-exclusion chromatography, performed using Superdex 200 10/300 GL column (Cytiva, Marlborough, MA, USA) equilibrated with the same buffer. The complex was concentrated and 5mM spermine was added just before the crystallization.

Crystallization of the SpOpB-TCK complex was started from the crystallization screening performed by the hanging drop vapor diffusion method on a robotic system (Rigaku Automation, Carlsbad, CA, USA). Ninety-six-well VDX plates (Hampton Research, Aliso Viejo, CA, USA) and crystallization screens CSHT, Index, PegION, PegRX, and TOP96 from Hampton Research and Anatrace, respectively, were used. The crystals were observed at 4 °C under two initial conditions: (1) 0.1 M MES 6.0, 20% propanol-2, and 20% PEG 2000MME; (2) 0.1 M imidazole pH 7.0 and 20% PEG 6000.

Optimization of crystal growth was performed using the same method and temperature in 24-well VDX plates (Hampton Research, Aliso Viejo, CA, USA). The best crystals were grown in 0.1M Imidazole, pH 7.0, and 25% PEG 6000. They reached their maximum size with an average length of the largest facet of about 200 μm during 3–4 weeks. Paraton was used for cryoprotection.

### 3.5. X-ray Diffraction and Structural Analysis

Diffraction data were collected at 100K at ID23-1 beamline (ESRF, Grenoble, France) [45]. The dataset was indexed, integrated, and scaled using the iMosflm package [46]. Space group was suggested by Pointless [47]. The diffraction data reported in Table 5 were obtained from a single crystal.

The structure was solved by the molecular replacement method using BALBES program [48]. The REFMAC5 program of the CCP4 suite [49] and the COOT interactive graphics program [50] were used for refinement and visual inspection of the electron density maps or manual rebuilding of the model, respectively.

$$*Rmrgd-F=2\sum_{hkl}\left|\langle I_{1}\left(hkl\right)\rangle -\langle I_{2}\left(hkl\right)\rangle \right|/\sum_{hkl}\langle I_{1}\left(hkl\right)\rangle -\langle I_{2}\left(hkl\right)\rangle$$

Visual inspection of the structure was performed using either PyMOL Molecular Graphics System Version 1.9.0.0 (Schrödinger, New York, NY, USA) or a COOT program [50]. Analysis of the interdomain and intradomain contacts was performed using either the COOT [50] or PDBePISA program [29]. Superpositions of structures were carried out using the PDBeFOLD program [51]. The Cα-superimpositions of the SpOpB-TCK structure (PDB ID 7YWP) on the SpOpBmod-TCK structure (PDB ID 7NE7) and SpOpB_S532A structure (PDB ID 7ZJZ), as well as on the SAXS validated SpOpB open state model and the TbOpB structures in closed and open conformations (PDB ID 4BP9 and 4BP8), were performed using the most conservative parts of the α/β hydrolase fold, which were determined by per-domain superimposition of the respective structures and consequent root mean square deviation (RMSD) analysis (Supplementary Figure S6 and Figure S7).The electrostatic charges were calculated using APBS Electrostatics Plugin for PyMol [52].

### 3.6. Data Bank Accession Numbers

The structure was deposited to the Protein Data Bank (PDB) under accession code (ID) 7YWP.

## 4. Conclusions

Oligopeptidase B is the least studied group in the POP family. Primarily this applies to the bacterial enzymes. Until recently, the structural studies of bacterial OpBs were far behind both the studies of protozoan OpBs and bacterial POPs. This situation began to change due to the appearance of first crystal structures of SpOpB. However, at first, there were only structures of the enzyme in an intermediate conformation with a disassembled catalytic triad, and this situation prevented the elucidation of the processes occurring in the active center during catalytic activation. Nevertheless, the obtained results served as the basis for further studies; in particular, the structure of the SpOpB open state was obtained using essential dynamics simulation and SAXS.

Here we described for the first time a crystal structure of SpOpB in the closed (catalytically active) conformation. The structure was obtained for the complex of the wild type enzyme with chloromethyl ketone inhibitor and transition state analog. TCK was simultaneously bound to the catalytic S532 and H652, imitating the tetrahedral complex, which allowed the suggestion of a scheme of SpOpB catalytic activation. The resulting structure, together with previously obtained structures of SpOpB in the intermediate and open states, as well as with known structures of protozoan TbOpB in the closed and open states, was used for comprehensive structural analysis. The analysis was focused on the relationship between the domains’ closure/opening and assembly/disassembly of the catalytic triad and on further stabilization of the catalytic triad in the closed state.

It was found that in SpOpB (as well as in TbOpB), the stabilization of the D-loop carrying the catalytic D, and, as a consequence, the entire catalytic triad, in a position favorable for the formation of a tetrahedral complex, occurs due to interaction with the neighboring loop from the β-propeller. However, it turned out that the mode of inter-domain interaction itself is significantly different for enzymes from bacteria and protozoa. In SpOpB, this interdomain interaction was based on the use of complementary oppositely charged surfaces with a low charge difference, while in TbOpB, SB1 was formed, which is a point electrostatic contact with a high charge difference. The choice of the type of interaction was determined by the nature of amino acid residues adjacent to the catalytic D and complementary residues from the β-propeller loop located opposite the D-loop. In SpOpB, it was a pair of polar residues (SQ) following the catalytic D617 and a pair of neighboring R residues from the β-propeller domain; in TbOpB, it was two oppositely charged residues, R and E, from the catalytic and β-propeller domains, respectively.

Bioinformatics analysis showed that based on these amino acid combinations: E/D-R (as in TbOpB) or RR-SQ (as in SpOpB), all bacterial OpBs can be divided into two large groups. The SpOpB-like group included 53% of bacterial enzymes, the TbOpB-like group included about 34%, and only 2% of proteins had other residues in these positions. What advantage this or that combination gives to the enzyme remains to be seen, but the direct dependence of the D-loop stabilization mode on the presence of a particular amino acid combination was confirmed by modeling the spatial structures of randomly selected OpBs belonging to the SpOpB- and TbOpB-like groups.

## Figures and Tables

**Table 1:** Comparison of the mutual arrangement of the catalytic triad residues and domains in the crystal structure of SpOpB-TCK with those in different states of SpOpB and TbOpB.

PDB ID or Source	7YWP	7ZJZ	Supplementary File S3	4BP8	4BP9
Conformation	closed	intermediate	open	open	closed
Protein [Ref]	SpOpB-TCK	SpOpB_S532A [23]	SpOpB-SAXS [23]	TbOpB [7]	TbOpB-AIP [7]
Residues in the structure	675	677	677	712	710
Aligned residues	-	670	539	527	668
Z-score *	-	51.7	43.1	48.5	45.4
Identity, % *	100	99	100	39	38
RMSD, Å *	-	1.7	3.8	3.4	1.5
Catalytic S–H Cα-distance, Å	7.9	17.9	17.4	18.5	8.3
Cat. S-OG Cat. H-NE2 distance, Å	2.8	n/a	21.3	18.3	3.5
Catalytic H–D Cα-distance, Å	4.6	7.2	6.0	7.6	4.5
Cat. H-ND1 Cat. D-OD2 distance, Å	3.0	6.6	8.8	11.8	3.1
Center of mass distance, Å	30.4	32.3	36.9	36.7	30.4
Buried surface area, cat/pro, % ^1^	16.8/13.8	11.7/9.8	8.8/7.3	8.4/7.5	14.0/12.3
Interface residues, cat/prop, % ^2^	21.5/18.8	15.7/15.3	11.6/10.9	10.3/10.5	17.4/16.9
Hydrogen bonds	24	15	12	14	28
Salt Bridges	4	2	2	3	4

*—according to Dali internet service. N/a—non-available due to the alanine substitution of the catalytic S532. ^1^—percentage of the buried surface area over the total surface area of the domain (according to PDBePisa). ^2^—percentage of residues in the interface over the total residues in the domain (according to PDBePisa).

**Table 2:** Polar contacts in the interface between the catalytic and β-propeller domains in the three states of SpOpB.

Structural Element		Closed		Intermediate		Open	
Propeller	Catalytic	Atom 1 prop.	Atom 2 cat.	Atom 1 prop.	Atom 2 cat.	Atom 1 prop.	Atom 2 cat.
Hinge1	α2	I71N	V68O	I71N	V68O		
	H-loop	P72O	R658NH2				
		Q73O	R658NH½				
β5/β6, Blade 1		E92O	R658NH1				
		N95O	R658NH1				
		E96O/OE2	R658N	E96O	R658NH2		
		Y97OH	S656OG	Y97OH	S656N		
				E96O	S656OG		
	H-loop, α12 border	E96OE2	F659N				
		E96OE2	K660N				
Blade 1/ Blade 2		E125O	K660NZ				
		*E125OE2*	*K660NZ*				
	H-loop	E125O	S656OG	A121O	K655NZ		
		Y127N	S656OG	R124O	K655NZ		
β9/β10, Blade 2		S149OG	G651N				
	α11	R150NH1	Q621OE1				
β13/β14, Blade 3	β2/α1	*K194NZ*	*D31OD2*				
Blade 3/ Blade 4	α8/α9	D222O	T574OG1				
β17/β18, Blade 4		T244OG1	G575O	T244OG1	D578OD2		
β21/β22, Blade 5	β35/α5					K291O	Q490NE2
	α5/α6	*K291NZ*	*E494OE½*	*K291NZ*	*E494OE½*	*K291NZ*	*E494OE1*
	β3/α5					N292ND2	L488O
		N292OD1	L490N	N292OD1	L490N	N292OD1	L490N
	α5					N292OD1	L491N
β24, Blade 6		M317SD	Q490N	M317SD	Q490N		
β25/β26, Blade 6	β34/α4					R333NH2	S458OG
		*R333NH½*	*D460OD½*	*R333NH½*	*D460OD½*	*R333NH½*	*D460OD½*
	β35/α5					R333NH1	E487O
	β32	G336O	R418NH2	G336O	R418NH2		
Blade 6/ Blade 7		T359OG1	R418NH1			D357OD1	S416OG
	β34/α4	T361N	P461O	T361N/OG1	P461O	T361N/OG1	P461O
β29/β30, Blade7		S380OG	F463N	S380OG	F463N	S380OG	F463N
	β33			M382SD	L433N		
Hinge2	α2	K407N	R70O	K407N	R70O	K407NE	R70O
						N408O	R70NH1
	η6	T410O	N413N	T410O/OG1	N413N/ND2	T410OG1	E412N

Salt bridges are in italic. H-bonds between different atoms of the same residue are highlighted in light grey, H-bonds between alternating residues are in grey.

**Table 3:** Polar contacts connecting D- and H-loops to each other and to other structural elements of the catalytic domain in the three states of SpOpB.

Conformations			Closed		Intermediate		Open	
Interacting Elements			Interacting Atoms					
1		2	1	2	1	2	1	2
H-loop	D-loop		M648O	H616NE2			M648O	H616ND1
			S650O	L615N				
			S650O	H616N			G651N	H616O
							D649O	D617N
			*H652ND1*	*D617OD*_1/2_			H652N	D617OD1
							G653N	D617OD1
							*R658NH*_1/2_	*D617OD*_1/2_
							R658NH1	S618OG
N-loop, β2/α1					R33NE	L615O		
							R33O	H616NE2
					R33NH2	D617O	R33NH2	Q619O
	α11		R33NH1	Q621OE1	R33NH2	Q621OE1	R33NH2	V620O
β36/α7			S532OG *	H652NE2 *				
β39	H-loop		Y645OH	K655NZ				
α12			E663OE2	K655NZ				
			D664OD2	K655N				
							Y662OH	F659O

Contacts inside the loops are excluded. Salt bridges are in italic. * In the 7YWP structure, the atoms are covalently bound to the inhibitor.

**Table 4:** Characteristics of four bacterial OpBs from causative agents of nosocomial infections belonging to the SpOpB- and TbOpB-like groups.

	Organism	Taxonomy ^1^	UniProt ID	Identity with SpOpB Sequence, % ^2^	RMSD Cα-Alignment on SpOpB Structure, Å ^3^	TbOpB or SpOpB-Like Group (Figure)
1	*Klebsiella* *pneumoniae*	Superphylum: Proteobacteria Class: Gammaproteobacteria Order: Enterobacteriales	W1DF13	65.63	0.6	SpOpB- like (Figure 9A)
2	*Pseudomonas* *aeruginosa*	Superphylum: Proteobacteria Class: Gammaproteobacteria Order: Pseudomonadales	Q9I440	42.81	1.3	TbOpB- like (Figure 9B)
3	*Stenotrophomonas* *maltophilia*	Superphylum: Proteobacteria Class: Gammaproteobacteria Order: Xanthomonadales	B2FHV9	50.67	1.1	SpOpB- like (Figure 9C)
4	*Mycobacterium* *tuberculosis*	Superphylum: Terrabacteria	O07178	23.97	2.9	TbOpB- like (Figure 9D)
5	*Trypanosoma* *Brucei* ^4^	Kingdom: Protozoa	O76728	38.10	1.5	Figure 8B

^1^ Only taxonomic subdivisions important for assignment to the SpOpB- and TbOpB-like group (see Figure 2) are shown. ^2^ According to BLAST ^3^ According to Dali internet service. ^4^ Included for comparison.

**Table 5:** Data collection, processing, and refinement.

PDB ID Protein-Inhibitor	7YWP SpOpB-TCK
Data collection	
Diffraction source	ESRF (ID23-1beamline)
Wavelength (Å)	0.98
Temperature (K)	100
Detector	PILATUS 6M
Space group	P2(1)2(1)2(1)
a, b, c (Å)	75.529, 89.660, 108.650
α, β, γ (°)	90.0
Unique reflections	37,524 (5487)
Resolution range (Å)	30.00–2.2 (2.32–2.2)
Completeness (%)	98.57 (99.90)
Average redundancy	4.14 (4.27)
〈I/σ(I)〉	6.6952 (3.89)
Rmrgd-F * (%)	7.8 (19)
Willson B	25.42
Refinement	
Rfact (%)	18.8
Rfree (%)	25.4
Rfree set size (%)	5
RMSD of bonds (Å)	0.008
RMSD of angles (°)	1.566
Ramachandran plot	
Most favored (%)	99.6
Allowed (%)	0.4
No. atoms	
Protein	5531
Water	417
Ligands	20
B-factor (Å2)	
Average	22.96
Protein	21.67
Water	26.42
Ligands	34.15

Values in parenthesis are for the highest-resolution shell.

## Data Availability

The structure was deposited to RCSB Protein Data Bank (https://www.rcsb.org/, accessed on 18 January 2023) under accession code 7YWP.

## Supplementary Materials

The following are available online at https://www.mdpi.com/article/10.3390/ijms24032286/s1.
