# Supplementary material for: Crystal Structure of Inhibitor-Bound Bacterial Oligopeptidase B in the Closed State: Similarity and Difference between Protozoan and Bacterial Enzymes
Source: Int J Mol Sci. 2023 Jan 24;24(3):2286. doi: 10.3390/ijms24032286 (PMC9917282; doi:10.3390/ijms24032286)
Supplement: Supplementary file 1 [file ijms-24-02286-s001.zip › Sup.FilesS1-S723.01.2022Edited.pdf]

Crystal structure of inhibitor-bound bacterial oligopeptidase B in the closed state: similarity and difference between protozoan and bacterial enzymes

Dmitry E. Petrenko[1,#], David M. Karlinsky[2,#], Veronika D. Gordeeva[3], Georgij P. Arapidi[2-4], Elena V. Britikova[5], Vladimir V. Britikov[5], Alena Y. Nikolaeva[1], Konstantin M. Boyko[6], Vladimir I. Timofeev[2,7], Inna P. Kuranova[7], Anna G. Mikhailova[2] , Eduard V. Bocharov[2,4], and Tatiana V. Rakitina[2,*].

1 National Research Center "Kurchatov Institute", 123182 Moscow, Russia
2 Shemyakin-Ovchinnikov Institute of Bioorganic Chemistry of the Russian Academy of Sciences, 117997 Moscow, Russia
3 Federal Research and Clinical Center of Physical-Chemical Medicine of Federal Medical Biological Agency, 119435 Moscow, Russia
4 Moscow Institute of Physics and Technology (national Research University), Phystech School of Biological and Medical Physics, 117303 Moscow, Russia
5 Institute of Bioorganic Chemistry of the National Academy of Sciences of Belarus, 220141 Minsk, Belarus
6 A.N. Bach Institute of Biochemistry, Federal Research Center of Biotechnology of the Russian Academy of Sciences, 119071 Moscow, Russia
7 Shubnikov Institute of Crystallography, Federal Scientific Research Centre "Crystallography and Photonics" of the Russian Academy of Sciences, 119333 Moscow, Russia
* Correspondence: taniarakitina@yahoo.com
† These authors contributed equally to this work

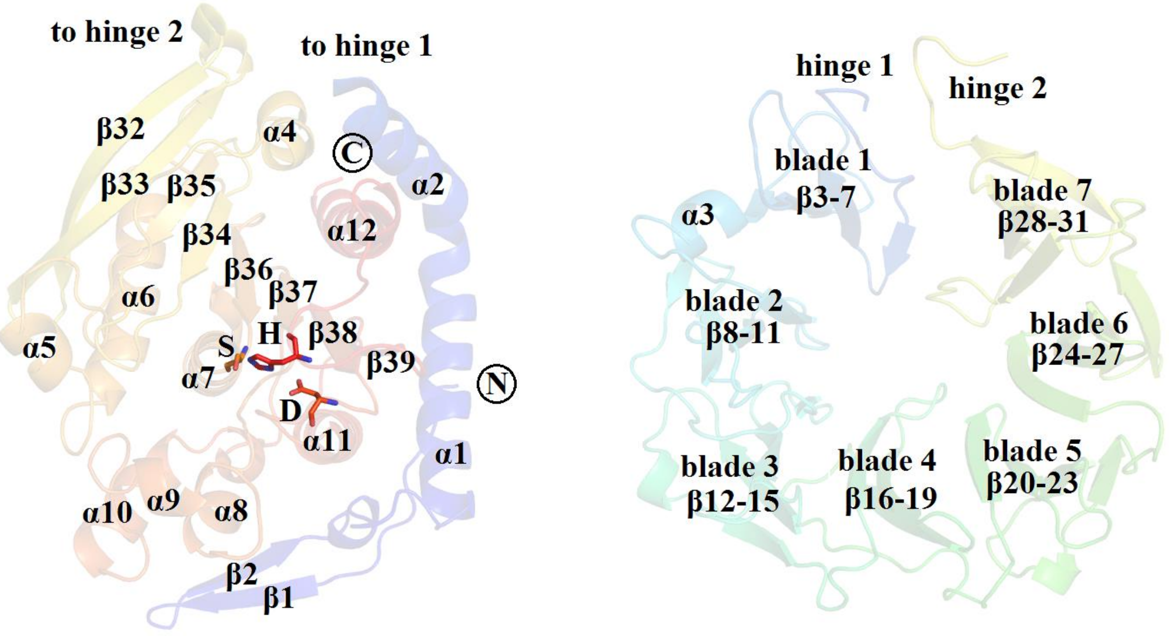

Supplementary Figure S1. Semitransparent cartoon presentations of the structures of two domains of SpOpB. The view is from inside of the interdomain cavity. The color coding is in rainbow style: the N-terminus (N) is in blue and the C-terminus is in red. The secondary structure elements and two hinge peptides are assigned. The catalytic domain (left) includes the N-terminal loop (blue) and the α/β hydrolase fold (from yellow to red), the catalytic triad residues (S, H, and D) are shown in sticks. The β-propeller domain (from blue to yellow) has the open-Velcro topology.

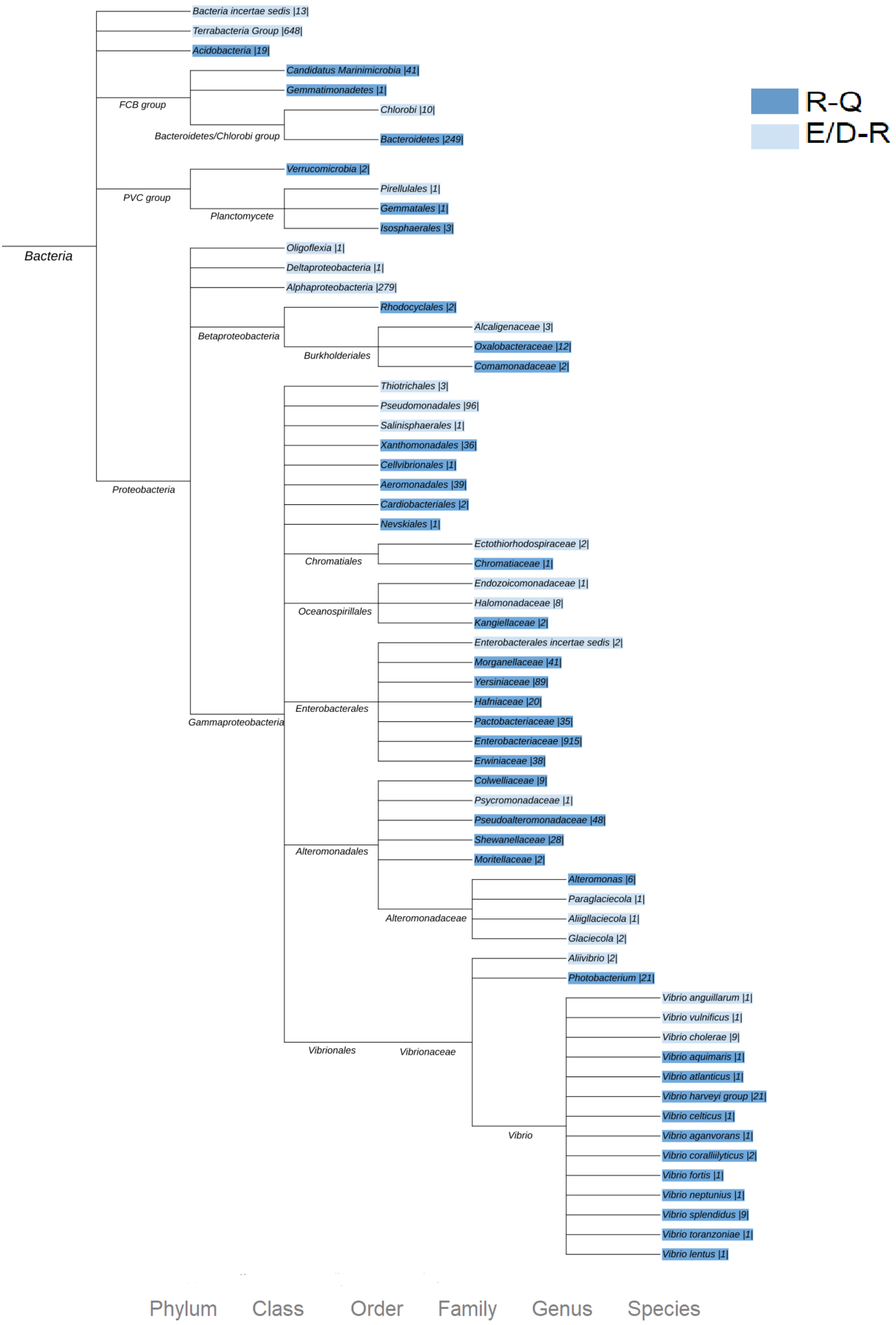

Supplementary Figure S2. Distribution of E/D-R and R-Q amino acid combinations over taxonomy tree. The number of sequences in the taxonomic group is indicated at the end of each branch. 2793 sequences were used in phylogenetic analysis.

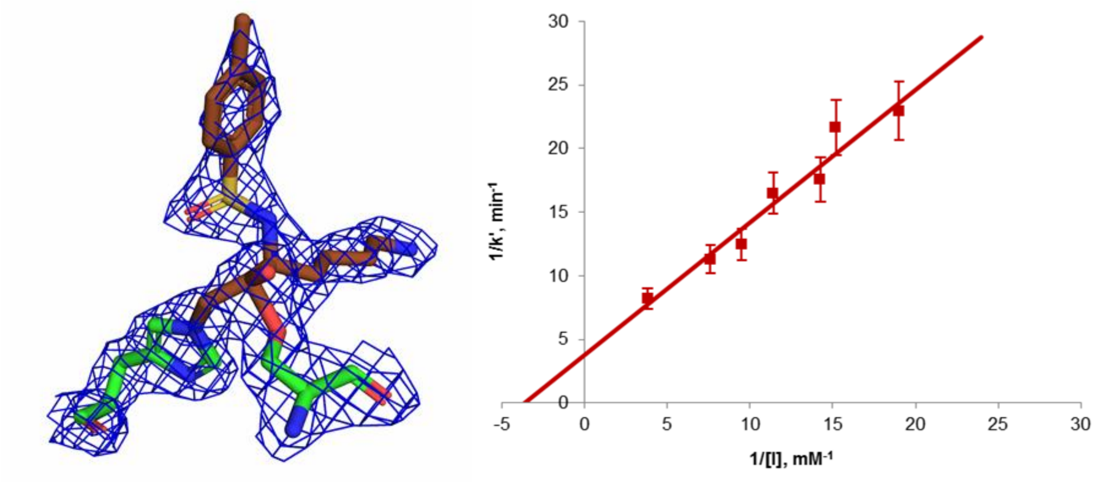

(**A**) (**B**)

Supplementary Figure S3. Interaction of the chloromethylketone inhibitor with SpOpB. **A**. The density maps of the TCK molecule (colored in brown) simultaneously bound to the catalytic S532 and H652 residues of SpOpB (colored in green). **B.** Inhibition of SpOpB (70 nM) by TCK in the concentration range 50–260 μM (0.1 M Tris-HCl buffer, pH 8.0, 25°C). The data was derived from at least 3 independent experiments.

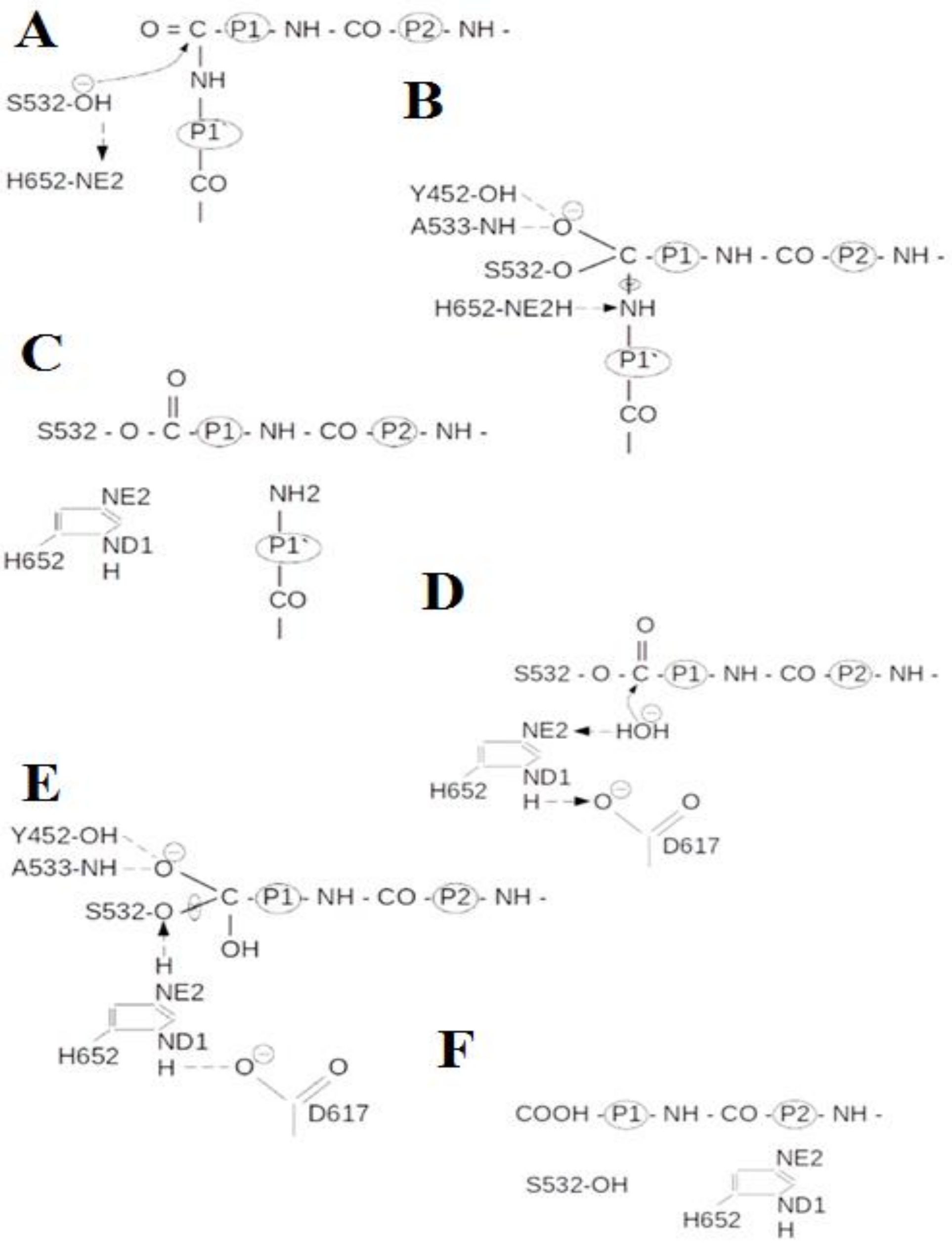

Supplementary Figure S4. Scheme of hydrolysis of a peptide substrate by SpOpB includes six steps: **A.** Enzyme-substrate (ES) complex formation: when H652 adopts its catalytical position between two other residues of the catalytic triad, it serves as a general base and its deprotonated NE2 atom accepts the H+ from the OH group of the catalytic S532. As a result, the nucleophile OG- of S532 attacks carbonyl C of the P1 residue of the substrate **B.** First oxyanion intermediate formation: when the resulting oxyanion (negatively charged tetrahedral intermediate) is stabilized in the oxyanion hole by two H-bonds, one with the main chain NH group of A533 and another with the OH group of Y452. An H-bond between protonated H652-NE2 and NH group of the substrate P1' residue promotes an H+ transfer. **C.** Acyl-enzyme intermediate formation: the H+ transfer leads to cleavage of the scissile C-N bond and collapse of the tetrahedral intermediate. The C-terminal part of the substrate is released, and the N-terminal part is still bound to S532 through acyl bond. **D.** Acyl-enzyme-H_2O complex formation: The carboxyl group of the catalytic D617 causes polarization of H652 by deprotonation of the ND1 atom of the imidazole ring, which causes another H^+ transfer from a water molecule to the NE2 atom of the imidazole ring. The

ionized H2O attacks the carbonyl C of the P1 residue. **E.** Second oxyanion intermediate formation: due to the attack, the carbonyl C returns to four-coordinated state (tetrahedral position) with the negative charge on the carbonyl O atom. **F.** Finally, the tetrahedral intermediate collapses freeing the N-terminal part of the substrate. The H^+ is transferred from the protonated NE2 atom of H532 to S532-OG. As a result, S532 and H652 return to their starting positions for the next catalytic cycle.

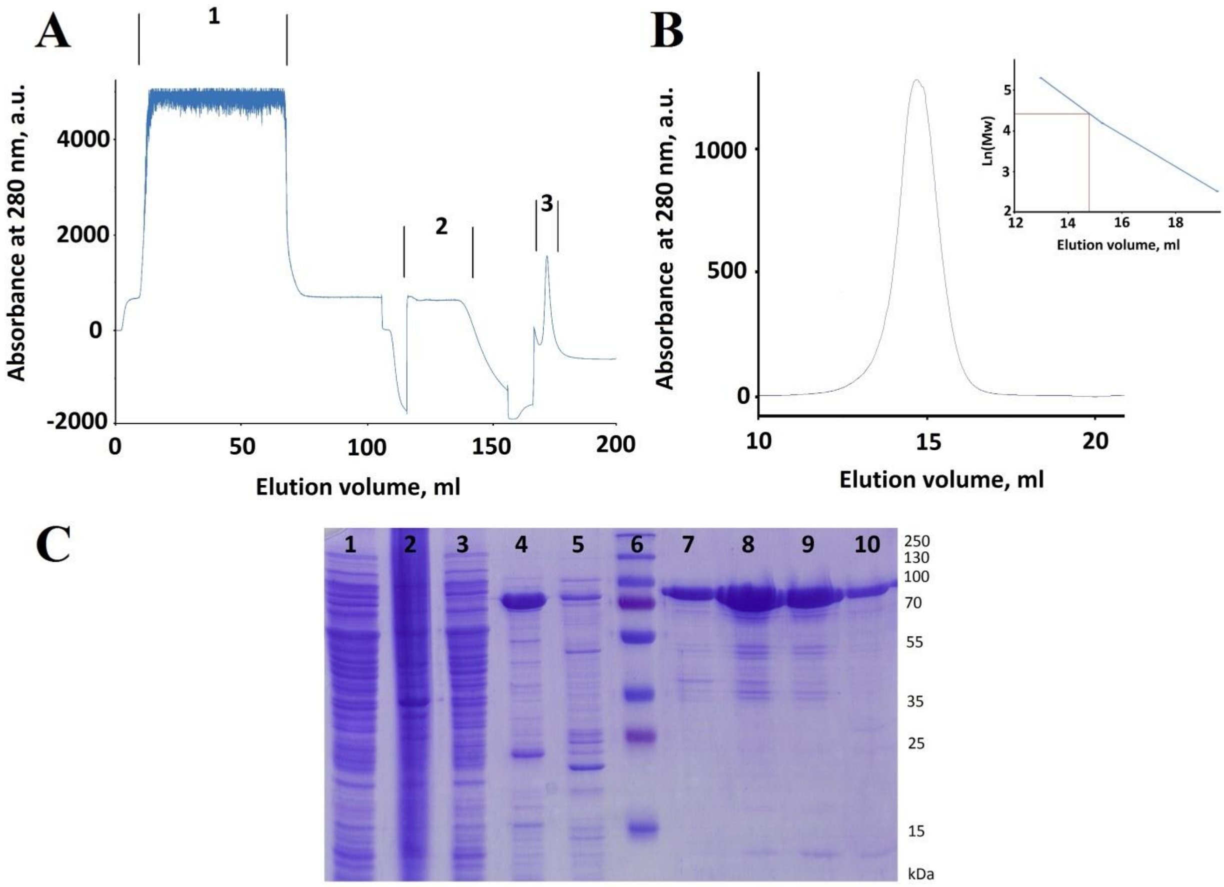

Supplementary Figure S5. Protein isolation and purification. **A.** Metal-chelate affinity chromatography: 1 – soluble components of bacterial cells not bound to the sorbent (leakage through the column); 2 – proteins washed off from the sorbent by the washing buffer; 3 – proteins washed off from the sorbent by the eluent buffer. **B.** Size-exclusion chromatography. **C**. SDS-PAGE of protein fractions: 1 – cell lysate; 2 – sediment; 3 – slip through the column; 4 – elution fraction from the Ni-NTA column; 5 – washing fraction from the Ni-NTA column, 6 – molecular weight marker (PageRuler™ Plus Prestained Protein Ladder), 7-10 – elution fraction from size-exclusion column.

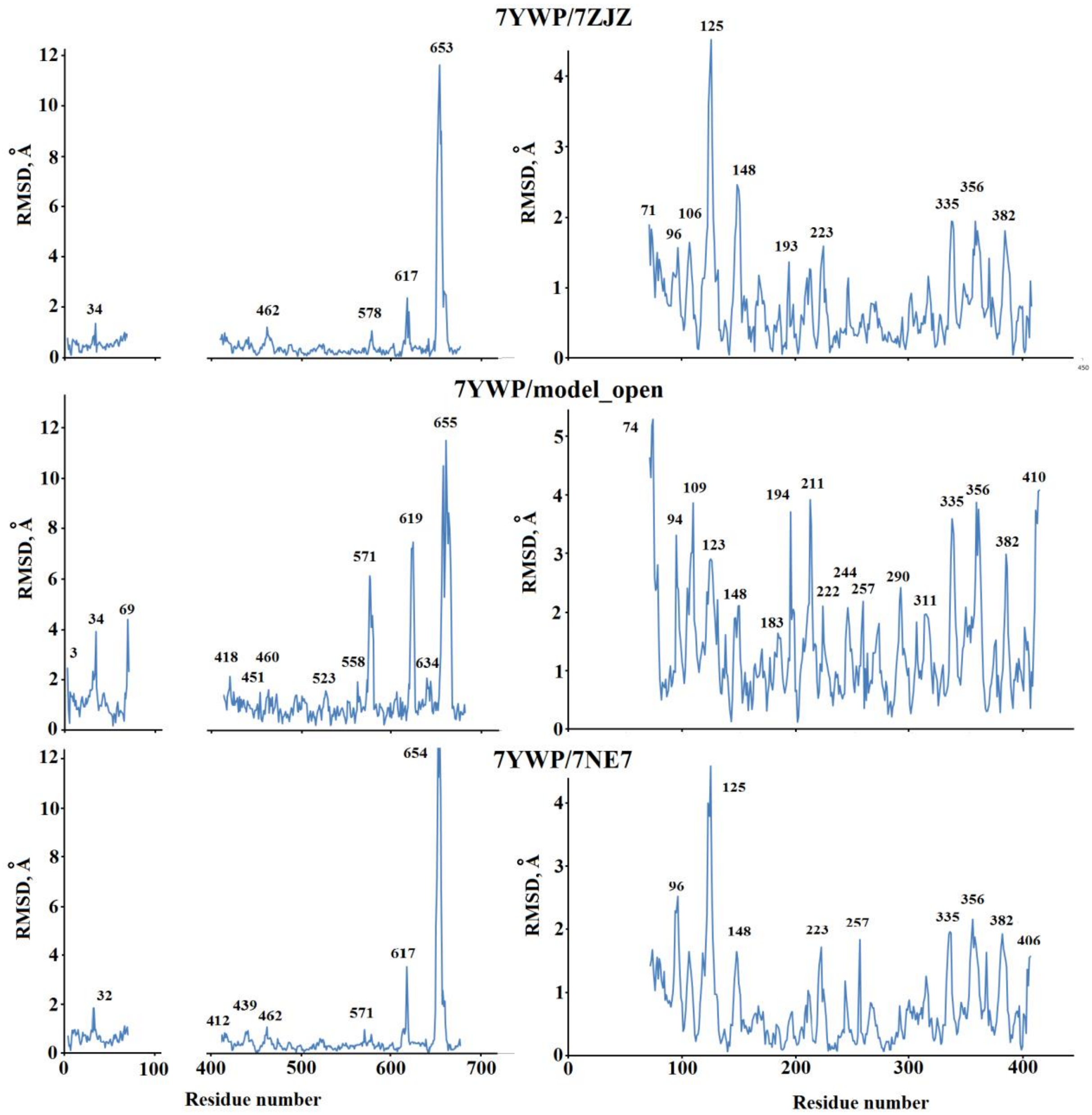

Supplementary Figure S6. Distributions of RMSD values along the SpOpB sequence. RMSD values were calculated upon Cα-atom superpositions of the individual domains: catalytic (left), β-propeller (right) of the respective (indicated above) structures.

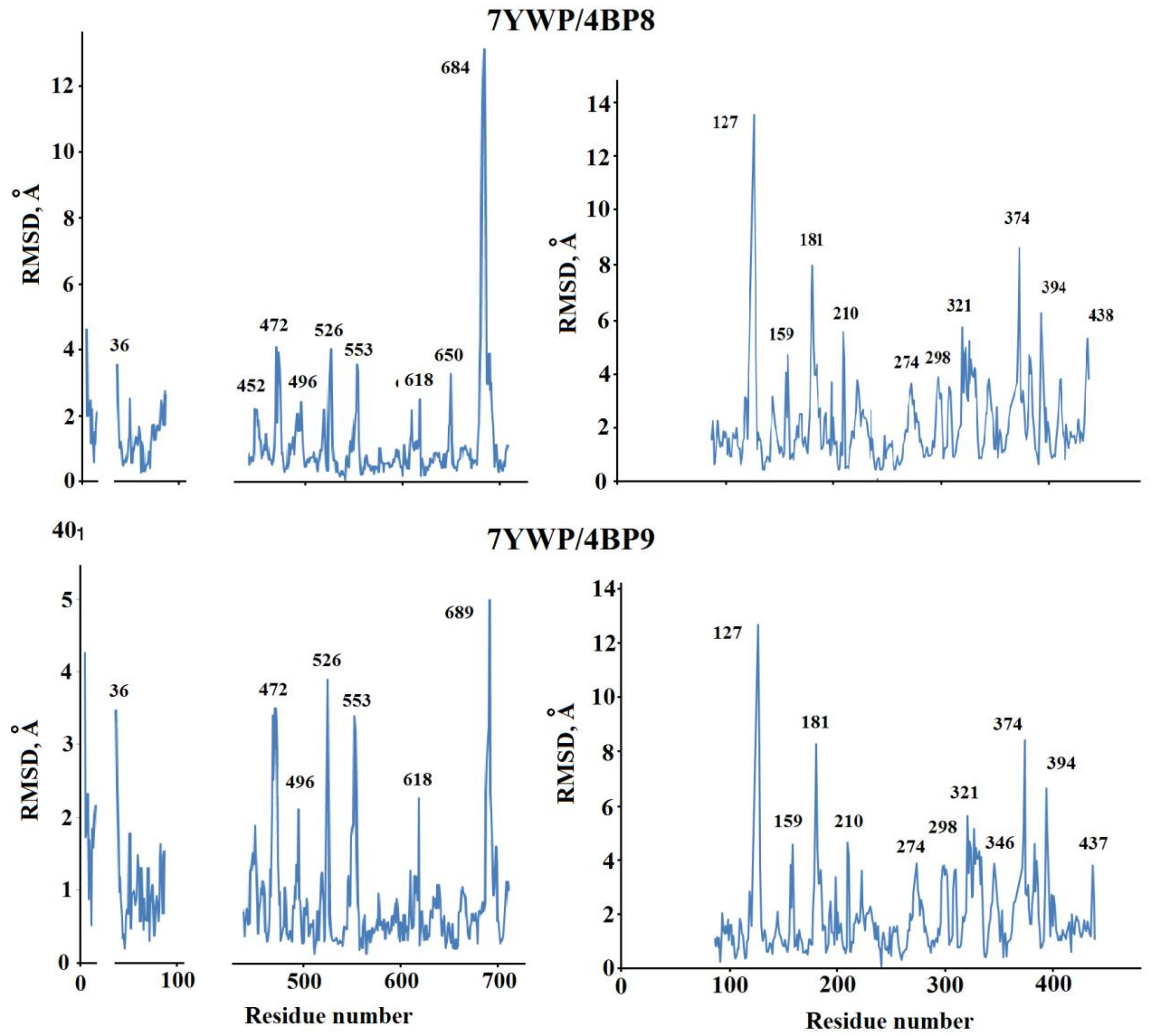

Supplementary Figure S7. Distributions of RMSD values along the TbOpB sequence. RMSD values were calculated upon Cα-atom superpositions of the individual domains: catalytic (left), β-propeller (right) of the respective (indicated above) structures.
